# Trends in diet structural composition and quality among adults in Beijing, China (2010–2022)

**DOI:** 10.3389/fnut.2025.1610823

**Published:** 2025-06-20

**Authors:** Ningsu Chen, Liyu Huang, Kai Zhao, Yimei Sha, Mengnan Zhao, Yihong Yao, Yana Qi, Yan Zhang, Bo Yu, Hairong He, Jiajie Yu, Jiali Duan

**Affiliations:** ^1^Department of Clinical Nutrition, Chinese Evidence-Based Medicine Center, West China Hospital, Sichuan University, Chengdu, China; ^2^Beijing Center for Disease Prevention and Control, Beijing, China

**Keywords:** dietary structure, diet quality, adult, trends, dietary composition

## Abstract

**Background:**

Unbalanced dietary patterns are a major risk factor for chronic non-communicable diseases. Examining trends in population-level dietary structural composition and quality is essential for monitoring nutritional transitions, identifying key dietary risks, and developing targeted public health policies.

**Methods:**

This study analyzed dietary trends among Beijing adults using three waves (2010–2022) of data from the China Nutrition and Health Surveillance (CNHS). Dietary intake was assessed through consecutive 3-day 24-h dietary recalls, supplemented by household weighing of cooking oils and condiments. Dietary structural composition was comprehensively analyzed, including the energy contribution of macronutrients and food sources of energy, protein, and fat intake. Population-level dietary quality was evaluated using the Chinese Diet Balance Index (DBI-22). Trend analyses were performed using the Jonckheere–Terpstra test, while group comparisons utilized chi-square and Mantel–Haenszel tests.

**Results:**

A total of 4,520 participants were included. Between 2010 and 2022, carbohydrate contribution to energy intake decreased from 56.1 to 46.7%, whereas fat intake increased from 31.6 to 36.9%. A dietary shift from plant-based to animal-sourced foods was observed, with the latter demonstrating annual increases in their proportional contributions to total energy, protein, and fat intake (*p* for trend <0.001). DBI-22 assessments revealed persistent imbalances relative to dietary guidelines, with insufficient intake of vegetables, fruits, dairy, aquatic products, and soybeans (median scores: −1.5 to −6) and excessive intake of cereals, meat, eggs, oil, and salt (median scores: 1 to 4.7). Trend analyses indicated a worsening in excessive intake (*p* = 0.001) alongside a modest improvement in intake deficiency (*p* = 0.004) over the study period. However, the overall gap between the actual and recommended intake remains unchanged (*p* = 0.868). Subgroup analyses revealed stable dietary transition trajectories across population strata, with significant interaction effects between area and time observed for both dietary composition and diet quality (*p* for interaction <0.05), indicating divergent trends between urban and rural residents over time.

**Conclusion:**

Between 2010 and 2022, Beijing adults experienced substantial imbalances in dietary structure, characterized by decreasing energy intake from carbohydrates and increasing intake from fat, both diverging further from recommended levels. Dietary deficiencies and excesses coexist, contributing to suboptimal dietary quality compared with national dietary guidelines.

## 1 Introduction

With rapid socioeconomic development and ongoing lifestyle changes, the dietary patterns of Chinese residents are undergoing significant transformations. This nutritional transition is marked by a shift from traditional plant-based diets—high in carbohydrates and dietary fiber—toward increasingly westernized diets characterized by greater consumption of animal-source foods and ultra-processed products. For instance, the average daily intake of ultra-processed foods in China rose from 12.6 grams in 1997 to 41.3 grams in 2011 (1). Concurrently, the proportion of energy derived from carbohydrates declined from 62.6% in 1991 to 50.6% in 2015, while the energy contribution from fat increased from 24.0 to 35.8% over the same period (2).

Such structural imbalances in dietary composition have been associated with elevated risks of overweight, obesity, cardiovascular diseases, type 2 diabetes, and other chronic non-communicable diseases (NCDs) (3, 4). According to the Global Burden of Disease Study 2017, dietary risk factors accounted for 11 million deaths and 255 million disability-adjusted life-years (DALYs) worldwide (5). Therefore, systematically monitoring the current status and temporal trends of population dietary patterns is essential for tracking nutritional transitions, identifying key dietary risks, and informing evidence-based nutrition policy interventions.

In addition to the overall composition of the diet, the balance of food group intake plays a critical role in shaping health outcomes. Previous studies have highlighted common dietary imbalances—such as excessive consumption of sodium and red meat, and inadequate intake of whole grains and fruits—as major contributors to adverse health outcomes (2). The Dietary Guidelines for Chinese Residents (2022) offer evidence-based recommendations to promote healthier national eating patterns, with a central emphasis on achieving dietary balance to prevent or mitigate diet-related chronic diseases (6). A balanced diet entails consuming a diverse range of food groups in appropriate proportions. Given the current nutritional profile of Chinese residents, the guidelines advocate for increased consumption of vegetables, fruits, whole grains, dairy products, and soy; moderate intake of poultry, red meat, fish, and eggs; and limited intake of cooking oil, salt, addible sugar, and alcohol. These guidelines serve as a critical reference for dietary assessment by quantifying gaps between actual intake and recommended levels, thereby facilitating the identification of dietary “deficiencies, excesses, and imbalances” and providing a comprehensive measure of diet quality.

The Dietary Balance Index (DBI)—a locally developed evaluation tool grounded in the Chinese dietary guidelines—captures both insufficient and excessive intake across multiple food groups, offering a multidimensional assessment of diet quality (7). The DBI has been extensively applied in national nutrition surveys and chronic disease research, demonstrating its utility and sensitivity in public health nutrition surveillance (8–10).

However, most existing studies on dietary nutrition in China are limited to cross-sectional analyses at single time points and lack systematic tracking of temporal dietary trends. Moreover, prior research often addresses either dietary composition or dietary quality in isolation, with few efforts to integrate these two complementary dimensions: macronutrient composition (i.e., energy contribution and food source structures of carbohydrates, fats, and proteins) and food group intake (e.g., grains, meats). These dimensions are inherently interconnected and provide mutually reinforcing insights into dietary patterns. As one of China’s most economically advanced and urbanized cities, Beijing serves as a valuable case for dietary surveillance. With 37.7% of its population comprising migrants, the city’s demographic diversity and rich food culture render it a microcosm of broader national trends (11). Assessing dietary shifts in this metropolitan setting can thus yield insights into nutritional transitions under rapid urbanization.

This study analyzes data from three waves (2010, 2015, and 2022) of the China Nutrition and Health Surveillance in the Beijing adult population, with two main objectives: (1) to evaluate changes in dietary composition, with a focus on the energy contribution of macronutrients (carbohydrates, fats, and proteins) and the evolution of their primary food sources among adult residents in Beijing; (2) to assess the distribution patterns and temporal trends of the DBI dimensions in accordance with the Dietary Guidelines for Chinese Residents (2022).

## 2 Methods

### 2.1 Data source and participants

This study utilized data from the China Nutrition and Health Surveillance (CNHS), a nationwide survey that provides comprehensive insights into dietary behaviors and chronic diseases. The CNHS was originally launched in 1959 as the National Nutrition Survey and was restructured in 2002 to establish the current framework, integrating nutrition and chronic disease surveillance components. The most recent round, conducted in 2022, represents the seventh iteration of the survey. The CNHS employs a stratified, multistage, random sampling method to select representative participants from 31 provincial regions across mainland China, accounting for both urban-rural divisions and population size (12).

In this study, we analyzed data from the 2010, 2015, and 2022 survey waves specific to Beijing. The number of surveillance sites in Beijing varied across these years: four in 2010, seven in 2015, and five in 2022. Site selection considered geographic and urban-rural stratification to ensure regional representativeness. Adults aged 18 years and older were included in the study, excluding individuals with incomplete dietary data as well as pregnant or lactating women. Ethical approval was obtained from the Ethics Committee of the National Institute of Nutrition and Health, Chinese Center for Disease Control and Prevention (Ethics numbers: 2022-008, 201519-A, 2013-018). Written informed consent was obtained from all participants.

### 2.2 Dietary evaluation

#### 2.2.1 Dietary intake assessment

Dietary intake was assessed through three consecutive 24-h dietary recalls, supplemented by household weighing of cooking oils and condiments. Individual consumption of oils and condiments was estimated proportionally to each family member’s meal consumption. This estimation was carried out through a two-step allocation process: first, each family member’s proportional contribution was determined based on their frequency of consuming home-prepared meals during the survey period and the proportion of each meal’s energy contribution to the total daily energy intake; second, these preliminary estimates were further adjusted using a relative coefficient derived from the ratio of each individual’s estimated energy requirement (EER) to the reference value of 2,150 kcal/day, consistent with the Chinese Dietary Reference Intakes (DRIs) for an 18-year-old male engaged in light physical activity (13). The detailed calculation of the allocation proportion for cooking oils and condiments refers to Equations (1)–(4). Total energy and nutrient intakes were primarily calculated using the China Food Composition Table (Standard Edition, 2018) (14). For food items not covered in this version, data were supplemented by the 2004 Edition (Volume 2) and 2009 Edition (Volume 1).


(1) $$\begin{array}{l} \text{Standard Person Coefficient}\\ =\text{Individual Estimated Energy Requirement}\;(\mathrm{EER})÷2,150\;\text{kcal} \end{array}$$

(2) $$\begin{array}{l} \text{Standard Person}-\text{Days}\\ =\text{Standard Person Coefficient}\times (\begin{array}{l} \beta \;\text{breakfast}\times n\;\text{breakfast}\\ +\beta \;\text{lunch}\times n\;\text{lunch}\\ +\beta \;\text{dinner}\times n\;\text{dinner} \end{array}) \end{array}$$

(3) $$\begin{array}{l} \text{Total Household Standard Person}-\text{Days}\\ =\sum (\text{Standard Person}-\text{Days of}\;\mathrm{All}\;\text{Members}) \end{array}$$

(4) $$\begin{array}{c} \text{Allocation Proportion}=\text{Standard Person}-\text{Days}\\ ÷\text{Total Household Standard Person}-\text{Days} \end{array}$$

Note: Estimated energy requirement (EER): The energy requirement determined based on factors such as age, sex, physiological status (including normal, pregnancy, and lactation), and physical activity level, as outlined in the Chinese Dietary Reference Intakes. Meal proportion (*β*): Energy proportion for each meal which is reported in surveys. Typically, the distribution is 30% for breakfast, 40% for lunch, and 30% for dinner. Meal frequency (*n*): Number of times an individual consumed a specific meal during the survey period.

#### 2.2.2 Dietary composition

Dietary structural composition was assessed by analyzing the distribution of energy from macronutrients and identifying key food sources contributing to energy, protein, and fat intake. Energy contributions from macronutrients are calculated using standard conversion factors: 4 kcal/g protein, 9 kcal/g fat, and 4 kcal/g carbohydrates (15). Macronutrient energy distribution was determined by dividing the energy contribution of each nutrient by total energy intake. Food sources were categorized based on established classification methods. Energy sources included cereals, soybeans, tubers and legumes, animal-based foods, cooking oils, edible sugar, alcoholic beverages and others. Protein sources were grouped into cereals, soybeans, animal-based foods, and others. Fat sources were classified as either animal-based or plant-based foods (16). The specific food items included in each food category are detailed in Supplementary Table 1.

#### 2.2.3 Diet quality

Overall dietary quality was assessed using the Chinese Diet Balance Index (DBI-22), developed in accordance with the 2022 Dietary Guidelines for Chinese Residents (7). The DBI-22 provides a comprehensive evaluation of dietary quality by scoring food intake as follows: a score of 0 indicates recommended intake, negative scores reflect insufficient intake, and positive scores indicate excessive intake. Three composite indicators were calculated:

- High Bound Score (HBS): Reflects excessive intake (1–9: acceptable; 10–18: mild excess; 19–27: moderate excess; ≥28: severe excess).
- Low Bound Score (LBS): Reflects insufficient intake (1–12: acceptable; 13–24: mild deficiency; 25–36: moderate deficiency; ≥37: severe deficiency).
- Diet Quality Distance (DQD): Measures overall deviation from recommended intake (1–17: acceptable; 18–34: mild imbalance; 35–50: moderate imbalance; ≥51: severe imbalance).

Detailed scoring methods have been described in the literature (7).

### 2.3 Statistical analysis

Categorical variables were described using frequency and percentage, whereas continuous data were presented as mean (standard deviation) or medians (interquartile range), depending on their distribution (normal or non-normal). Missing data in categorical baseline characteristics (marital status and educational level, 2.4% missing rate) were imputed using the mode. Due to the skewed distribution of dietary intake data identified in normality tests, temporal trends were assessed using the Jonckheere–Terpstra test (a non-parametric method for detecting monotonic trends in ordinal time data, suitable for non-normal distributions), with the Benjamini–Hochberg (BH) method applied to adjust *p*-values for multiple comparisons across time points or subgroups. Subgroup analyses were conducted to explore potential differences in dietary structure and dietary quality across various subgroups, including age, sex, and region. For each subgroup, temporal trends were examined using the Jonckheere–Terpstra test. Additionally, the interaction between subgroup characteristics and time was assessed using analysis of variance (ANOVA) to evaluate how different factors influenced the temporal changes. Group comparisons utilized chi-square and Mantel–Haenszel tests. All analyses were conducted using R (version 4.4.1) using a two-sided significance level of *α* = 0.05.

## 3 Results

### 3.1 Participant characteristics

**Figure 1:**
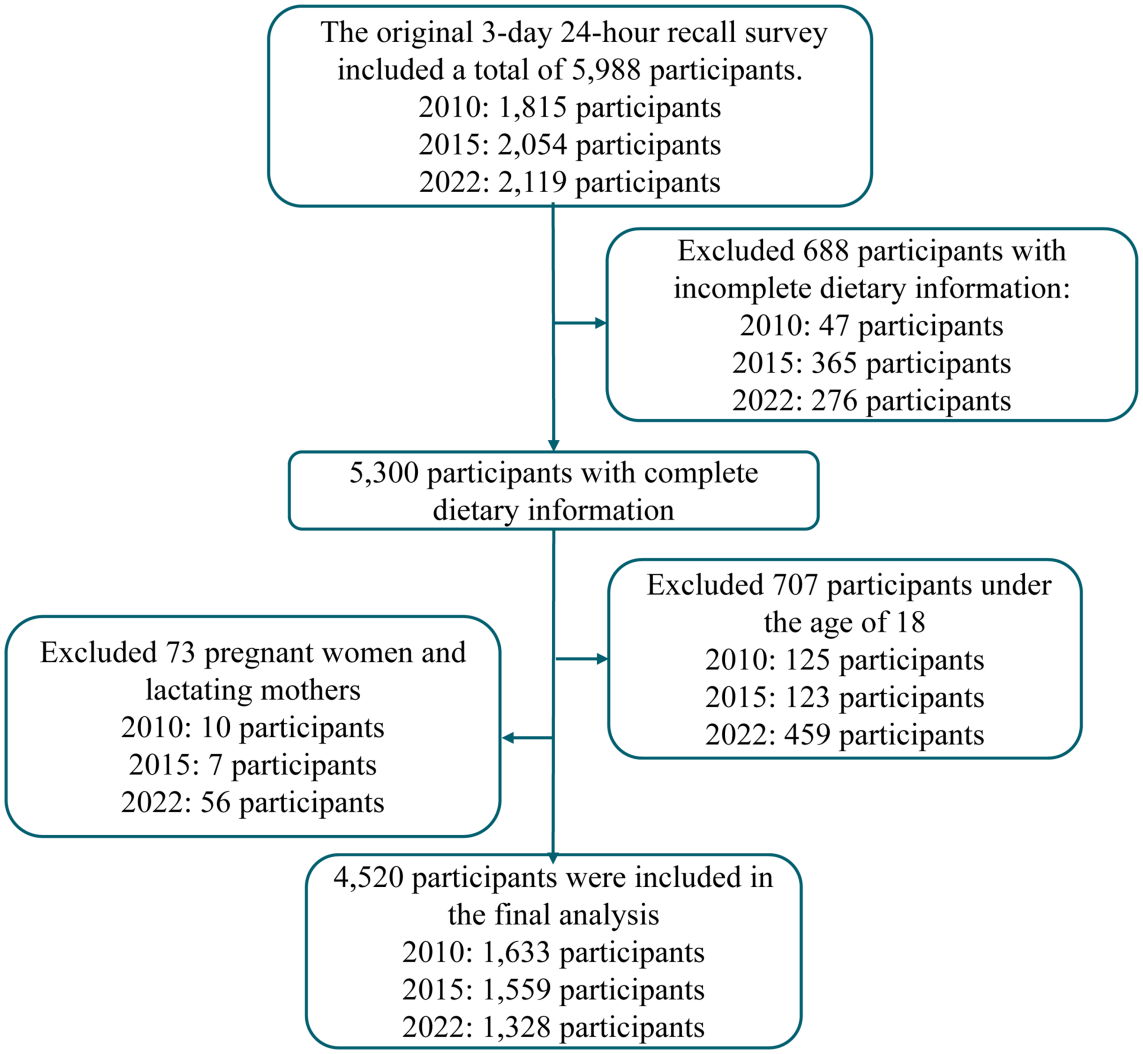
Flow diagram of study participant selection.

A total of 4,520 participants were included across three survey waves: 1,633 in 2010, 1,559 in 2015, and 1,328 in 2022 (Figure 1). The mean age was 53.8 years with specific distribution as follows: 28.3% were 18–44 years old, 34.4% were 45–59 years old, and 37.3% were ≥60 years old. Males accounted for 45.8% of participants, and 52.5% resided in urban areas. 88.5% were currently married and 25.1% received college education or above (Table 1).

**Table 1:** Basic characteristics of the study participants.

Variables	Total *N* = 4,520	2010 *N*1 = 1,633	2015 *N*2 = 1,559	2022 *N*3 = 1,328	*p*
Gender, *n* (%)					0.809
Male	2068 (45.8)	754 (46.2)	703 (45.1)	611 (46.0)	
Female	2,452 (54.2)	879 (53.8)	856 (54.9)	717 (54.0)	
Age, *n* (%)					**<0.001**
18–44	1,281 (28.3)	408 (25.0)	364 (23.3)	509 (38.3)	
45–59	1,555 (34.4)	618 (37.8)	577 (37.0)	360 (27.1)	
≥60	1,684 (37.3)	607 (37.2)	618 (39.6)	459 (34.6)	
Area, *n* (%)					**<0.001**
Urban	2,373 (52.5)	884 (54.1)	703 (45.1)	786 (59.2)	
Rural	2,147 (47.5)	749 (45.9)	856 (54.9)	542 (40.8)	
Marital status, *n* (%)					**<0.001**
Married	4,000 (88.5)	1,378 (84.4)	1,449 (92.9)	1,173 (88.3)	
Never married	226 (5.0)	119 (7.3)	36 (2.3)	71 (5.3)	
Divorced	79 (1.7)	32 (2.0)	19 (1.2)	28 (2.1)	
Widowed	215 (4.8)	104 (6.4)	55 (3.5)	56 (4.2)	
Educational levels, *n* (%)					**<0.001**
Primary or below	795 (17.6)	352 (21.6)	319 (20.5)	124 (9.3)	
Junior high school	1,526 (33.8)	482 (29.5)	610 (39.1)	434 (32.7)	
High school/vocational school	1,066 (23.6)	416 (25.5)	361 (23.2)	289 (21.8)	
College or higher	1,133 (25.1)	383 (23.5)	269 (17.3)	481 (36.2)	

Bold values in the last column indicate statistical significance with *p* < 0.05.

### 3.2 Dietary structure

As shown in Table 2, the overall median energy contribution among Beijing adults was 52.7% from carbohydrates, 33.5% from fat and 12.8% from protein. Between 2010 and 2022, the proportion of energy from carbohydrates significantly decreased from 56.1 to 46.7% (*p* < 0.001), while energy from fat (31.6 to 36.9%) and protein (12.0 to 14.9%) both increased significantly (*p* < 0.001). Cereals remained the primary energy source (47.8%), followed by animal-based foods (17.4%) and cooking oils (14.0%). Tubers/legumes (1.2%) and soybeans (0.7%) contributed minimally to energy intake. Over time, energy derived from soybeans, animal-based foods, and cooking oils increased, while that from cereals, tubers, legumes, sugars, and alcoholic beverages declined.

**Table 2:** Trends in the dietary structural composition of adults in Beijing (%).

Variables	Total *N* = 4,520	2010 *N*1 = 1,633	2015 *N*2 = 1,559	2022 *N*3 = 1,328	*p* for trend
Percentage of energy from nutrients [median (IQR)]					
Carbohydrate	52.7 (45.4, 60.6)	56.1 (48.5, 64.1)	54.4 (47.9, 60.9)	46.7 (40.5, 54.2)	**<0.001**
Fat	33.5 (26.7, 40.7)	31.6 (24.6, 38.8)	33.0 (26.7, 39.1)	36.9 (29.4, 43.9)	**<0.001**
Protein	12.8 (11.0, 15.0)	12.0 (10.6, 14.0)	12.3 (10.8, 13.9)	14.9 (12.8, 17.5)	**<0.001**
Energy sources by food groups [median (IQR)]					
Cereals	47.8 (37.5, 58.6)	49.3 (38.1, 61.9)	50.2 (41.5, 59.3)	42.2 (33.1, 52.6)	**<0.001**
Soybeans	0.7 (0.0, 2.4)	0.5 (0.0, 2.0)	0.5 (0.0, 2.1)	1.1 (0.0, 3.3)	**<0.001**
Tubers and legumes	1.2 (0.0, 3.4)	1.6 (0.0, 4.1)	1.1 (0.0, 3.4)	1.0 (0.0, 2.5)	**<0.001**
Animal-based foods	17.4 (10.1, 25.6)	13.4 (6.8, 21.2)	16.7 (10.1, 23.8)	23.9 (15.4, 32.1)	**<0.001**
Cooking oils	14.0 (9.0, 20.6)	13.2 (8.7, 19.7)	14.2 (9.5, 20.2)	14.6 (8.8, 22.3)	**0.002**
Edible sugar	0.0 (0.0, 0.6)	0.0 (0.0, 0.8)	0.0 (0.0, 0.5)	0.0 (0.0, 0.6)	**0.010**
Alcoholic beverages	0.0 (0.0, 0.0)	0.0 (0.0, 0.0)	0.0 (0.0, 0.0)	0.0 (0.0, 0.0)	**<0.001**
Others	9.6 (5.4, 15.8)	12.3 (6.4, 20.0)	8.7 (5.3, 13.7)	8.5 (4.8, 13.6)	**<0.001**
Protein sources by food groups [median (IQR)]					
Cereals	41.2 (29.0, 54.8)	43.8 (30.5, 58.1)	46.7 (34.9, 58.3)	31.7 (23.6, 44.1)	**<0.001**
Soybeans	2.0 (0.0, 7.3)	1.7 (0.0, 7.0)	2.0 (0.0, 7.3)	2.3 (0.0, 7.4)	0.121
Animal-based foods	36.3 (22.8, 49.4)	29.2 (16.5, 42.9)	34.5 (22.8, 45.9)	48.0 (35.0, 59.6)	**<0.001**
Others	13.6 (9.2, 20.1)	18.1 (12.4, 25.7)	12.3 (8.3, 17.4)	11.4 (7.9, 15.6)	**<0.001**
Fat sources by food groups [median (IQR)]					
Animal-based foods	33.9 (20.1, 48.1)	27.8 (14.4, 41.5)	34.5 (21.6, 47.0)	41.6 (26.5, 56.8)	**<0.001**
Plant-based foods	66.1 (51.9, 79.9)	72.2 (58.5, 85.6)	65.5 (53.0, 78.4)	58.4 (43.2, 73.5)	**<0.001**

Bold values in the last column indicate statistical significance with *p* < 0.05.

Cereals (41.2%) and animal-based foods (36.3%) were the two major sources of protein. From 2010 to 2022, protein from cereals decreased from 43.8 to 31.7% (−12.1 percentage points), while protein from animal-based foods increased from 29.2 to 48.0% (+18.8 percentage points) (both *p* < 0.001). Although plant-based sources still accounted for the majority of fat intake (66.1%), the proportion of animal-derived fat increased significantly from 27.8 to 41.6% (*p* < 0.001).

Stratified analyses by age, gender, and geographic region showed trends consistent with those in the overall population, with declining carbohydrate intake and increasing fat and protein intake observed across all subgroups. Animal-based contributions to energy, protein, and fat intake increased universally, whereas plant-based contributions declined.

Significant interactions between geographic region and survey year were detected for all dietary structure indicators except for alcohol-derived energy (*p* < 0.05), indicating divergent trends between urban and suburban populations. Specifically, energy derived from edible sugar declined overall but increased slightly in urban areas, while protein from soybeans remained stable overall but decreased in suburban areas and increased in urban areas (*p* for interaction <0.05). The subgroup results are provided in Supplementary Table 2.

### 3.3 Diet quality

As shown in Table 3, DBI-22 score analysis showed a significant increase in High Bound Score (HBS) (*p* = 0.001) and a significant decrease in LBS (*p* = 0.004), indicating increased dietary excess but improved intake adequacy. However, no statistically significant change was observed in the Diet Quality Distance (DQD) (*p* = 0.868).

**Table 3:** Trends in DBI-22 scores among adults in Beijing.

DBI-22 scores	Total *N* = 4,520	2010 *N*1 = 1,633	2015 *N*2 = 1,559	2022 *N*3 = 1,328	*p* for trend
Indicators for evaluating inadequate intake——dietary guidelines recommend increasing consumption [median (IQR)]					
Vegetables	−1.5 (−2.7, 0.0)	−1.6 (−2.7, −1.0)	−1.4 (−2.6, 0.0)	−1.6 (−2.7, 0.0)	0.882
Fruits	−2.5 (−6.0, −1.0)	−1.9 (−6.0, −0.4)	−1.9 (−6.0, −0.4)	−6.0 (−6.0, −2.3)	**<0.001**
Dairy	−6.0 (−6.0, −3.6)	−6.0 (−6.0, −3.6)	−6.0 (−6.0, −3.8)	−6.0 (−6.0, −3.6)	0.064
Aquatic products	−4.0 (−4.0, −3.0)	−4.0 (−4.0, −3.0)	−4.0 (−4.0, −3.0)	−4.0 (−4.0, −2.0)	**<0.001**
Soybeans	−3.7 (−6.0, −0.6)	−3.8 (−6.0, −0.6)	−3.9 (−6.0, −1.2)	−3.6 (−6.0, 0.0)	0.064
Indicators for evaluating moderate intake——dietary guidelines recommend maintaining moderate consumption [median (IQR)]					
Cereals	4.7 (0.0, 12.0)	5.9 (0.4, 12.0)	4.7 (0.4, 9.6)	3.5 (0.0, 9.8)	**<0.001**
Meat	2.0 (−1.0, 4.0)	0.0 (−2.0, 3.0)	1.0 (−1.0, 4.0)	4.0 (1.0, 4.0)	**<0.001**
Eggs	0.0 (−2.0, 2.0)	−1.0 (−3.0, 1.0)	0.0 (−2.0, 2.0)	1.0 (−1.0, 4.0)	**<0.001**
Indicators for evaluating excessive intake——dietary guidelines recommend reducing consumption [median (IQR)]					
Cooking oils	1.0 (0.0, 3.0)	1.0 (0.0, 2.8)	1.0 (0.0, 3.2)	1.0 (0.0, 3.4)	**0.004**
Addible sugar	0.0 (0.0, 0.0)	0.0 (0.0, 0.0)	0.0 (0.0, 0.0)	0.0 (0.0, 0.0)	0.565
Salt	2.0 (1.0, 3.7)	2.3 (1.0, 4.1)	1.9 (1.0, 3.3)	1.9 (1.0, 4.0)	**0.001**
Alcoholic beverage	0.0 (0.0, 0.0)	0.0 (0.0, 0.0)	0.0 (0.0, 0.0)	0.0 (0.0, 0.0)	0.194
Dietary diversity and overall score [median (IQR)]					
Dietary variety	−5.0 (−6.0, −4.0)	−5.0 (−7.0, −4.0)	−5.0 (−6.0, −4.0)	−5.0 (−6.0, −3.0)	**<0.001**
HBS	13.0 (9.1, 16.7)	13.0 (8.8, 16.8)	12.0 (8.8, 15.6)	13.7 (9.6, 17.6)	**0.001**
LBS	23.4 (18.2, 28.8)	23.9 (17.9, 29.8)	23.0 (18.1, 28.6)	23.2 (18.5, 27.9)	**0.004**
DQD	36.9 (28.6, 44.0)	37.5 (28.2, 45.6)	36.1 (28.3, 42.7)	37.6 (29.6, 44.0)	0.868

Bold values in the last column indicate statistical significance with *p* < 0.05.

Inadequate consumption persisted for vegetables, fruits, dairy, aquatic products, and soybeans (median LBS: −1.5 to −6.0), while excessive intake was observed for cereals, meat, cooking oils, and salt (median HBS: 1.0 to 4.7). Although aquatic product consumption increased significantly (*p* < 0.001), deficits in vegetable and dairy intake remained stable (*p* = 0.882, 0.064, respectively), and fruit insufficiency worsened (median score declined from −1.9 to −6.0; *p* < 0.001). Excessive consumption of cereals and salt declined, whereas meat, eggs, and cooking oil overconsumption increased. Dietary variety improved significantly (*p* < 0.001), yet remained suboptimal.

Stratified analyses by age, gender, and region largely mirrored the overall trends, showing decreased intake of fruits and cereals, increased intake of aquatic products, meat, and eggs, and improved dietary diversity. However, several subgroup deviations were observed (Supplementary Table 3). Significant age-time interactions were identified for dairy, meat, and alcohol intake (*p* for interaction <0.05), while gender-time interactions were found for fruit and alcohol consumption (*p* for interaction <0.05).

Urban-rural disparities were pronounced across several indicators. Urban residents had lower dietary inadequacy scores (for all indicators of inadequate intake except vegetables, *p* < 0.05) and greater dietary diversity (median −4.0 vs. −6.0, *p* < 0.001), lower excess intake of cereals (median 2.2 vs. 8.3, *p* < 0.001) and salt (1.8 vs. 2.4, *p* < 0.001), but higher meat intake (3.0 vs. 0.0, *p* < 0.001). Temporal trends revealed a decline in urban dairy intake but an improvement in rural consumption, whereas soybean intake exhibited the reverse trend. Region-time interactions were significant for all indicators except sugar, alcohol, and dietary variety (*p* for interaction <0.001).

Age-specific trends showed that the 45–59 age group had the poorest diet quality (median DQD: 38.0 vs. 36.5/36.4, *p* = 0.005). Excessive intake (HBS) worsened among older adults (≥60 years), while no significant changes occurred in younger adults (18–44). Women had better diet quality than men (DQD: 36.5 vs. 37.4, *p* = 0.004). Over time, LBS declined among men, while HBS increased among women, although no significant gender-time or age-time interactions were detected for HBS, LBS, or DQD (all *p* for interaction >0.05). Compared to rural residents, urban populations exhibited lower excessive intake (HBS 10.7 vs. 14.6, *p* < 0.001), reduced inadequacy (LBS 20.0 vs. 27.1, *p* < 0.001), and better dietary balance (DQD 31.1 vs. 42.1, *p* < 0.001). Importantly, DQD improved over time in rural areas but worsened in urban populations, with significant region-time interactions observed for all three DBI indicators (*p* for interaction <0.001).

### 3.4 Diet quality by sociodemographic characteristics

**Figure 2:**
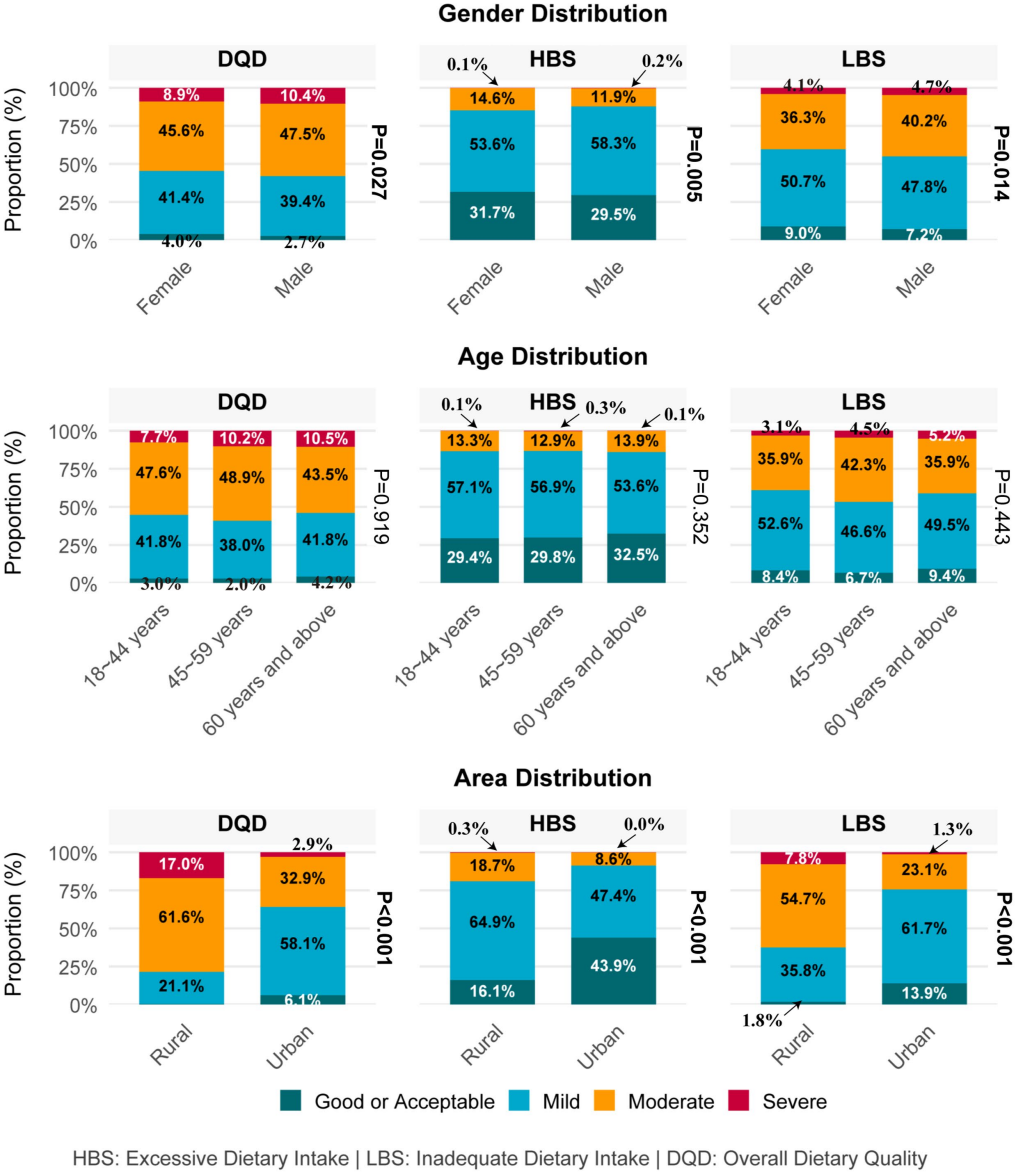
Dietary quality of adults in Beijing across different characteristics. (Some color blocks are barely visible due to their extremely small proportions, and their proportional values are indicated by arrows).

Females demonstrated better dietary quality than males, as shown by higher proportions of “good/acceptable” ratings for DQD (4.0% vs. 2.7%) and lower rates of moderate/severe dietary imbalance (45.6%/8.9% vs. 47.5%/10.4%). Similar, females had better HBS (31.7% vs. 29.5%) and LBS (9.0% vs. 7.2%) scores, indicating less excessive and inadequate intake compared to males. Urban residents showed better dietary quality than rural counterparts, with significantly higher “good/acceptable” rates for HBS (43.9% vs. 16.1%), LBS (13.9% vs. 1.9%), and DQD (6.1% vs. 0.4%). No significant age-related differences were observed in the HBS, LBS, or DQD classifications (*p* = 0.352, 0.443, 0.919), details are presented in Figure 2 and Supplementary Table 4.

## 4 Discussion

This study shows a significant transformation in the dietary composition of adults in Beijing from 2010 to 2022. The median energy contribution from fat increased to 33.5%, exceeding the recommended upper limit of 30%, and further increased to 36.9% by 2022. Meanwhile, the proportion of energy from carbohydrates declined steadily from 56.1% in 2010 to 46.7% in 2022, below the recommended lower threshold of 50%. Although plant-based foods remain the main sources of dietary energy, protein, and fat, the contribution of animal-based foods has increased significantly. In 2022, animal foods accounted for 23.9% of total energy intake, 48.0% of protein intake and 41.6% of fat intake. This changes suggest a shift from a traditional plant-based dietary pattern to one characterized by higher intakes of fat and animal-based foods. Similar shifts was observed in China between 1982 and 2012, with carbohydrate intake decreasing from 80.8 to 55.9%, while fat intake rose from 12.0 to 32.3% (17). This was accompanied by reduced consumption of whole grains and vegetables, alongside an increased intake of red and processed meats, which have been associated with adverse health outcomes such as obesity, hypertension, and increased cardiometabolic risk (17).

Importantly, health outcomes are influenced not only by the quantity but also by the quality of macronutrients, defined by their specific food sources. Accumulating evidence suggests that different food sources of dietary fat, protein, and carbohydrates result in different health outcomes (18). Plant-based sources, such as grains, vegetables, legumes, and nuts, are associated with lower risks of non-communicable diseases and mortality, whereas animal-based sources from meat, dairy products, and eggs are correlated with increased risks (19, 20). These findings highlight the need to enhance both the quantity and quality of the diet by reducing total fat intake while improving the quality of fat and protein sources through increased consumption of plant-based foods.

The dietary quality assessment using DBI-22 scores further illustrates a dual burden of dietary inadequacy and excess. While excessive intake of cereals, meat, cooking oils, and salt persists, insufficient consumption of vegetables, fruits, dairy, aquatic products, and soybeans remains widespread. Although the intake of aquatic products has increased significantly, deficits in vegetables and dairy products have shown little improvement, and fruit intake has worsened. The Lower Bound Score (LBS), reflecting inadequate intake, showed only modest improvement, while the Higher Bound Score (HBS), indicating excessive intake, worsened over time. Overall, the Dietary Quality Distance (DQD) remained unchanged, suggesting persistent dietary imbalance. Additionally, dietary variety remains limited, with a median intake of only 7 out of 12 recommended food categories. These patterns are consistent with global findings. A 2019 study published in The Lancet analyzing dietary risks across 195 countries found widespread inadequacies: average global intakes of nuts/seeds, dairy, and whole grains reached only 12, 16, and 23% of recommended levels, respectively. Meanwhile, consumption of sodium, processed meat, and red meat significantly exceeded optimal levels by 86, 90, and 18%, respectively (5). These imbalances contribute to an increased risk of chronic diseases, including cardiovascular disease and cancer (21–24).

Subgroup analyses revealed relatively stable trends over time, with significant disparities across sociodemographic strata. Among age and sex subgroups, though fluctuations in macronutrient composition and DBI-22 scores were observed, statistically significant time-subgroup interactions were identified for only a minority of indicators. Women exhibited better dietary quality compared to men. While both younger adults (18–44 years) and older adults (≥60 years) demonstrated lower DQD scores than middle-aged adults (45–59 years), these differences were not statistically significant when categorized by diet quality classification. Urban-rural differences were more pronounced, with significant region-time interactions for most dietary indicators. In terms of dietary composition, urban residents showed a pronounced increase in fat intake and a higher contribution of animal-based macronutrients, whereas rural diets retained traditionally higher proportions of plant-based sources. DBI-22 assessments revealed that urban residents had less severe dietary insufficiencies compared to rural counterparts. However, excess intake patterns differed regionally: rural residents more often exceeded recommended levels for grains and salt, while urban residents showed higher overconsumption of meat. Notably, urban populations scored more favorably on the DQD, HBS, and LBS, with a significantly greater proportion classified as having “Good or Acceptable” dietary quality. These findings suggest a more balanced dietary pattern in urban residents, characterized by reduced extremes of overconsumption and deficiency.

These findings are supported by studies in other countries. A cohort study conducted in the United States demonstrated that rural residents had a 61% higher risk of poor dietary quality than urban dwellers (25). Similarly, global research has consistently shown that women tend to have better dietary quality, with gender gaps in Alternative Healthy Eating Index (AHEI) scores ranging from 2.1 to 5.0 points (26). These differences may be explained by higher nutritional literacy and greater access to diverse foods in urban areas, as well as women’s generally greater health awareness and lower preference for high-fat diets (27, 28). The dietary transitions observed in this study may be driven by economic development. Previous studies have shown that increasing disposable income leads to a shift in dietary preferences, away from carbohydrates and towards higher consumption of animal-based foods. It is estimated that for every 1% increase in income, fat and protein intake will increase by 0.12 and 0.11%, respectively (29). Moreover, the increasing popularity of food delivery and eating out may negatively impact dietary quality, as these habits are associated with larger portion sizes and higher intake of energy-dense foods, but lower intake of whole grains, fruits, and vegetables (30).

These findings highlight the need for targeted nutrition interventions. For rural areas, efforts should focus on nutrition education, reducing grain and salt overconsumption, and improving access to healthy foods through food supply optimization and subsidies. In urban areas, interventions should prioritize reducing meat and edible sugar intake and promoting plant-based alternatives. Gender-specific approaches should focus on men, emphasizing increased consumption of fruits and dairy products while reducing excessive intake of salt and oil through tailored health education. Promoting dietary diversity and balancing both the quantity and quality of dietary intake are critical for all populations.

This study has several limitations. First, its geographical focus on Beijing limits the generalizability of findings to other regions in China. Second, the reliance on 3-day 24-h dietary recall may not fully capture the long-term habitual dietary intake and is subject to recall bias. Third, the analysis focused exclusively on the food sources of macronutrients (e.g., plant-based vs. animal-based fats/proteins) and dietary intake balance but did not assess specific nutrient intakes (e.g., vitamins, minerals, micronutrients). This limits our understanding of how dietary quality relates to nutrient adequacy, representing an important future research direction. Fourth, although the sample was selected via multistage stratified random sampling, some imbalance in baseline characteristics (e.g., age) was observed across the three waves. While subgroup analyses demonstrated consistent trends across demographic groups, this imbalance, combined with the regional focus on Beijing, warrants caution in extrapolating results to broader populations or national contexts. Future research could consider developing mobile-based, real-time dietary assessment tools using image recognition and portion estimation technologies to reduce reliance on participant recall and improve data accuracy in dietary surveys. Additionally, longitudinal studies with nationwide sampling and comprehensive nutrient intake analyses would strengthen the generalizability and mechanistic understanding of dietary patterns.

## 5 Conclusion

This study analysed dietary data from three waves (2010–2022) of the China Nutrition and Health Surveillance, a nationally representative survey, to examine trends among adults aged 18 years and older in Beijing. The findings show a persistent dietary imbalance, characterized by an increasing proportion of energy intake from fat and a decreasing proportion from carbohydrates. Moreover, deficiencies in the intake of vegetables, fruits, and dairy products coexist with excessive consumption of cereals, oils, and salt, which deviate substantially from national dietary guidelines. These findings highlight the urgent need for strengthened public nutrition education and targeted interventions to promote balanced, health-promoting dietary patterns.

## Data Availability

Data will be made available on reasonable request. Requests to access these datasets should be directed to yujiajie@wchscu.edu.cn.

## Supplementary material

Supplementary Table 1 Food items included in each food category.

Supplementary Table 2 Trends in dietary structural composition among Beijing adults by demographic characteristics.

Supplementary Table 3 Trends in DBI-22 Scores among Beijing adults by demographic characteristics.

Supplementary Table 4 Dietary quality of adults in Beijing across different characteristics, n(%).
