# Supplementary material for: Trends in diet structural composition and quality among adults in Beijing, China (2010–2022)
Source: Front Nutr. 2025 Jun 20;12:1610823. doi: 10.3389/fnut.2025.1610823 (PMC12226269; doi:10.3389/fnut.2025.1610823)
Supplement: Supplementary Table 1 — Food items included in each food category. [file Table_1.docx]

**Supplementary Table 1** Food items included in each food category

|  | **Food Group** | **Food Items** |
| --- | --- | --- |
| **Food Groups of Energy Sources** | **Cereals** | Noodles, steamed bun (mantou), layered steamed bun (huajuan), flatbread (dabing), other wheat flour products; rice (cooked rice), corn, millet, sorghum, buckwheat, oats, etc. |
|  | **Soybean** | soybeans: yellow soybeans, black soybeans, green soybeans, etc. soybean products: tofu, tofu skin, dried tofu, soy milk, etc. |
|  | **Tubers and Legumes** | Tubers: potato, sweet potato, etc. legumes: mung beans, red beans, pinto beans, kidney beans, peas, fava beans, etc. |
|  | **Animal-based Foods** | Meat: Pork, ham, beef, lamb, pork floss, sausage, offal (animal organs), chicken, duck, etc. Dairy: Cow milk, milk powder, camel milk, goat milk, cheese, yogurt, etc. Eggs: Chicken eggs, duck eggs, goose eggs, etc. Aquatic products: Fish, shrimp, crab, scallops, clams, squid, etc. |
|  | **Cooking Oils** | Animal fats: Beef tallow, lard, butter, ghee, etc. Vegetable oils: Rapeseed oil, soybean oil, peanut oil, corn oil, olive oil, etc. |
|  | **Edible Sugar** | White sugar, rock sugar, brown sugar, maltose, chocolate candies, fruit candies, honey, etc. |
|  | **Alcoholic Beverages** | Baijiu (Chinese liquor), wine, huangjiu (Chinese rice wine), whiskey, vodka, cocktails, etc. |
|  | **Others** | Vegetables: Radish, green beans, chili peppers, eggplant, pumpkin, napa cabbage, etc. Fungi & Algae: Shiitake, enoki mushrooms, white fungus, black fungus, tea tree mushrooms, kelp, nori, etc. Fruits: Apples, pears, grapes, bananas, watermelon, etc. Nuts: Walnuts, almonds, pine nuts, chestnuts, peanuts, sunflower seeds, etc. Snacks: Rice crust (guoba), potato chips, crackers, packaged bread, etc. Beverages: Lemon soda, cola, pineapple juice drinks, orange juice drinks, tea, etc. |
| **Food Groups of Protein Sources** | **Cereals** | Same as the "Food Groups of Energy Sources" listed above |
|  | **Soybean** | Same as the "Food Groups of Energy Sources" listed above |
|  | **Animal-based Foods** | Same as the "Food Groups of Energy Sources" listed above, with the addition of animal fats such as beef tallow, lard, butter, ghee, etc.,since there is no separate cooking oil category. |
|  | **Others** | Vegetable oils, Vegetables,Fungi & Algae,Fruits,Nuts,Snacks,Beverages, etc. |
| **Food Groups of Fat Sources** | **Animal-based Foods** | Same as the "Food Groups of Protein Sources" listed above |
|  | **Plant-based foods** | Cereals, soybeans, vegetable oils, vegetables, fungi & algae, fruits, nuts, beverages, and other non-animal foods |

**Supplementary Table 2** Trends in dietary structural composition among Beijing adults by demographic characteristics

|  | **Age** | | |  |  | **Gender** | |  |  | **Area** | |  |  |
| --- | --- | --- | --- | --- | --- | --- | --- | --- | --- | --- | --- | --- | --- |
|  | **18~44 years** | **45~59 years** | **60 years and above** | **P values** | **p-value for interaction** | **Female** | **Male** | **P values** | **p-value for interaction** | **Rural** | **Urban** | **P values** | **p-value for interaction** |
| **n** | 1,281 | 1,555 | 1,684 |  |  | 2,452 | 2,068 |  |  | 2,147 | 2,373 |  |  |
| **Percentage of energy from nutrients (median (IQR))** | | | | | | | | | | | | | |
| **Carbohydrate** | 50.0 (43.1, 58.0)↓ | 53.7 (45.4, 61.4)↓ | 53.9 (47.3, 61.8)↓ | **<0.001** | 0.167 | 53.1 (46.0, 60.8)↓ | 52.3 (44.5, 60.1)↓ | **0.003** | 0.302 | 56.0 (48.0, 64.0)↓ | 50.3 (43.6, 57.3)↓ | **<0.001** | **<0.001** |
| **Fat** | 35.6 (28.2, 42.4)↑ | 33.3 (26.3, 40.9)↑ | 32.5 (25.7, 39.4)↑ | **<0.001** | 0.052 | 33.3 (26.6, 40.3)↑ | 33.8 (26.8, 41.1)↑ | 0.218 | 0.383 | 31.4 (24.3, 38.9)↑ | 35.4 (28.9, 41.7)↑ | **<0.001** | **<0.001** |
| **Protein** | 13.5 (11.3, 16.1)↑ | 12.4 (10.8, 14.3)↑ | 12.7 (10.9, 15.0)↑ | **<0.001** | **0.019** | 12.8 (11.0, 15.0)↑ | 12.9 (11.1, 15.1)↑ | 0.338 | 0.192 | 11.7 (10.3, 13.7)↑ | 13.8 (11.9, 16.0)↑ | **<0.001** | **<0.001** |
| **Energy Sources by Food Groups (median (IQR))** | | | | | | | | | | | | | |
| **Cereals** | 44.9 (34.3, 55.3)↓ | 48.9 (38.7, 59.8)↓ | 49.0 (38.6, 59.8) | **<0.001** | 0.084 | 47.5 (37.2, 58.3)↓ | 48.0 (37.8, 59.0)↓ | 0.256 | 0.28 | 53.9 (44.6, 65.3)↓ | 41.9 (33.4, 51.9)↓ | **<0.001** | **<0.001** |
| **Soybeans** | 0.8 (0.0, 2.6)↑ | 0.6 (0.0, 2.4)↑ | 0.5 (0.0, 2.4)↑ | **0.036** | 0.269 | 0.6 (0.0, 2.4)↑ | 0.7 (0.0, 2.5)↑ | 0.167 | 0.562 | 0.0 (0.0, 1.8) | 1.1 (0.0, 2.9)↑ | **<0.001** | **<0.001** |
| **Tubers and legumes** | 1.2 (0.0, 3.0)↓ | 1.1 (0.0, 3.3)↓ | 1.4 (0.0, 3.8)↓ | **0.044** | **0.022** | 1.3 (0.0, 3.6)↓ | 1.1 (0.0, 3.2)↓ | 0.050 | 0.695 | 1.3 (0.0, 3.6)↓ | 1.2 (0.0, 3.2)↓ | 0.585 | **0.026** |
| **Animal-based foods** | 20.2 (12.7, 29.0)↑ | 15.7 (9.0, 24.0)↑ | 16.6 (9.6, 24.7)↑ | **<0.001** | 0.449 | 17.3 (10.2, 25.5)↑ | 17.5 (10.0, 25.6)↑ | 0.943 | 0.208 | 12.9 (6.6, 20.7)↑ | 21.0 (14.2, 28.9)↑ | **<0.001** | **<0.001** |
| **Cooking oils** | 13.8 (8.4, 21.7)↑ | 14.8 (9.7, 21.2)↑ | 13.5 (8.7, 19.3) | **<0.001** | **0.001** | 14.0 (8.9, 20.6) | 14.0 (9.1, 20.6)↑ | 0.844 | 0.496 | 14.8 (9.5, 21.7) | 13.3 (8.7, 19.4)↑ | **<0.001** | **<0.001** |
| **Edible sugar** | 0.0 (0.0, 0.7) | 0.0 (0.0, 0.6)↓ | 0.0 (0.0, 0.6)↓ | **0.002** | 0.695 | 0.0 (0.0, 0.6)↓ | 0.0 (0.0, 0.6)↓ | 0.887 | 0.651 | 0.0 (0.0, 0.0)↓ | 0.0 (0.0, 0.8)↑ | **<0.001** | **0.012** |
| **Alcoholic beverages** | 0.0 (0.0, 0.0)↓ | 0.0 (0.0, 0.0)↓ | 0.0 (0.0, 0.0)↓ | **<0.001** | **0.003** | 0.0 (0.0, 0.0) | 0.0 (0.0, 0.0)↓ | **<0.001** | **<0.001** | 0.0 (0.0, 0.0)↓ | 0.0 (0.0, 0.0)↓ | 0.956 | 0.208 |
| **Others** | 9.9 (5.4, 16.2)↓ | 9.0 (5.2, 15.2)↓ | 9.7 (5.5, 16.0)↓ | **0.102** | 0.803 | 10.1 (5.9, 16.5)↓ | 8.8 (5.0, 15.0)↓ | **<0.001** | **0.019** | 7.4 (4.5, 12.4)↓ | 11.9 (6.9, 18.6)↓ | **<0.001** | **<0.001** |
| **Protein Sources by Food Groups (median (IQR))** | | | | | | | | | | | | | |
| **Cereals** | 35.4 (25.3, 49.6)↓ | 44.0 (31.9, 57.6)↓ | 43.1 (30.2, 56.0)↓ | **<0.001** | 0.214 | 40.8 (28.7, 54.8)↓ | 41.7 (29.5, 54.8)↓ | 0.206 | 0.362 | 50.8 (39.0, 62.7)↓ | 33.0 (24.6, 45.0)↓ | **<0.001** | **<0.001** |
| **Soybean** | 2.3 (0.0, 7.3) | 1.9 (0.0, 7.3) | 1.8 (0.0, 7.2) | 0.168 | 0.728 | 1.8 (0.0, 7.1) | 2.2 (0.0, 7.5) | 0.133 | 0.803 | 0.0 (0.0, 6.4)↓ | 3.1 (0.0, 8.0)↑ | **<0.001** | **<0.001** |
| **Animal-based foods** | 42.7 (28.3, 55.4)↑ | 33.8 (20.7, 46.7)↑ | 34.5 (21.3, 47.2)↑ | **<0.001** | 0.214 | 36.2 (22.8, 49.3)↑ | 36.3 (22.9, 49.4)↑ | 0.99 | 0.280 | 28.1 (16.0, 41.8)↑ | 42.9 (31.0, 54.0)↑ | **<0.001** | **<0.001** |
| **Others** | 12.6 (8.7, 18.9)↓ | 13.9 (9.3, 20.0)↓ | 14.1 (9.6, 21.0)↓ | **<0.001** | **0.011** | 14.1 (9.6, 20.7)↓ | 12.8 (8.8, 19.6)↓ | **<0.001** | 0.739 | 12.9 (8.8, 19.4)↓ | 14.1 (9.6, 20.6)↓ | **<0.001** | **<0.001** |
| **Fat Sources by Food Groups (median (IQR))** | | | | | | | | | | | | | |
| **Animal-based foods** | 36.9 (22.9, 51.5)↑ | 31.3 (18.1, 45.4)↑ | 34.0 (19.2, 48.2)↑ | **<0.001** | 0.132 | 33.7 (19.9, 48.0)↑ | 34.1 (20.3, 48.2)↑ | 0.586 | 0.192 | 28.3 (14.6, 43.7)↑ | 38.3 (25.6, 50.9)↑ | **<0.001** | **<0.001** |
| **Plant-based foods** | 63.1 (48.5, 77.1)↓ | 68.7 (54.6, 81.9)↓ | 66.0 (51.8, 80.8)↓ | **<0.001** | 0.132 | 66.3 (52.0, 80.1)↓ | 65.9 (51.8, 79.7)↓ | 0.586 | 0.192 | 71.7 (56.3, 85.4)↓ | 61.7 (49.1, 74.4)↓ | **<0.001** | **<0.001** |

**Note:** Upward arrows indicate that the indicator shows an increasing trend over time, downward arrows indicate a decreasing trend over time, and no arrow indicates that the temporal trend test for the indicator was not statistically significant. P-values represent the results of the difference test, with bolded values indicating statistical significance. P-values for interaction are derived from analysis of variance (ANOVA) examining the interaction between subgroup characteristics and time; bolded values indicate statistically significant interactions.

**Supplementary Table 3** Trends in DBI-22 Scores among Beijing adults by demographic characteristics

| **DBI-22** | **Age** | | |  |  | **Gender** | |  |  | **Area** | |  |  |
| --- | --- | --- | --- | --- | --- | --- | --- | --- | --- | --- | --- | --- | --- |
|  | **18-44 years** | **45-59 years** | **60 years and above** | **P values** | **p-value for interaction** | **Female** | **Male** | **P values** | **p-value for interaction** | **Rural** | **Urban** | **P values** | **p-value for interaction** |
| **n** | 1,281 | 1,555 | 1,684 |  |  | 2,452 | 2,068 |  |  | 2,147 | 2,373 |  |  |
| **Indicators for evaluating inadequate intake——dietary guidelines recommend increasing consumption [median (IQR)]** | | | | | | | | | | | | | |
| **Vegetable** | -1.7 (-2.8, -1.0) | -1.5 (-2.7, -1.0) | -1.3 (-2.5, 0.0) | **<0.001** | 0.355 | -1.4 (-2.6, 0.0) | -1.7 (-2.7, -1.0) | **<0.001** | 0.237 | -1.5 (-2.6, 0.0) | -1.5 (-2.7, 0.0) | 0.531 | **0.006** |
| **Fruit** | -6.0 (-6.0, -1.0)↓ | -2.5 (-6.0, -1.0)↓ | -2.3 (-6.0, -0.7)↓ | **0.001** | 0.517 | -2.1 (-6.0, -0.5)↓ | -6.0 (-6.0, -1.2)↓ | **<0.001** | **0.003** | -6.0 (-6.0, -1.6)↓ | -1.7 (-6.0, -0.2)↓ | **<0.001** | **<0.001** |
| **Dairy** | -6.0 (-6.0, -3.6↑ | -6.0 (-6.0, -3.8)↑ | -6.0 (-6.0, -3.3)↓ | **<0.001** | **0.040** | -6.0 (-6.0, -3.6) | -6.0 (-6.0, -3.6)↑ | **0.001** | 0.794 | -6.0 (-6.0, -6.0)↑ | -4.1 (-6.0, -2.7)↓ | **<0.001** | **<0.001** |
| **Aquatic products** | -4.0 (-4.0, -2.0)↑ | -4.0 (-4.0, -3.0)↑ | -4.0 (-4.0, -3.0)↑ | **<0.001** | 0.884 | -4.0 (-4.0, -3.0)↑ | -4.0 (-4.0, -3.0)↑ | 0.682 | 0.873 | -4.0 (-4.0, -4.0)↑ | -4.0 (-4.0, -2.0)↑ | **<0.001** | **<0.001** |
| **Soybean** | -3.6 (-6.0, -0.4) | -3.8 (-6.0, -0.6) | -3.9 (-6.0, -0.6) | 0.082 | 0.355 | -3.8 (-6.0, -0.5) | -3.7 (-6.0, -0.7) | 0.362 | 0.828 | -6.0 (-6.0, -1.6)↓ | -3.1 (-6.0, 0.0)↑ | **<0.001** | **<0.001** |
| **Indicators for evaluating excessive intake——dietary guidelines recommend reducing consumption [median (IQR)]** | | | | | | | | | | | | | |
| **Cereals** | 4.0 (0.0, 9.6)↓ | 4.7 (0.2, 12.0)↓ | 5.4 (0.2, 12.0) | **<0.001** | 0.106 | 5.4 (0.1, 12.0)↓ | 4.1 (0.0, 9.6)↓ | **<0.001** | 0.145 | 8.3 (3.2, 12.0)↓ | 2.2 (0.0, 6.9)↓ | **<0.001** | **<0.001** |
| **Meat** | 4.0 (0.0, 4.0)↑ | 1.0 (-1.0, 4.0)↑ | 1.0 (-1.0, 4.0)↑ | **<0.001** | **0.001** | 2.0 (-1.0, 4.0)↑ | 2.0 (-1.0, 4.0)↑ | 0.209 | 0.568 | 0.0 (-2.0, 4.0)↑ | 3.0 (0.0, 4.0)↑ | **<0.001** | **<0.001** |
| **Eggs** | 0.0 (-2.0, 3.0)↑ | 0.0 (-2.0, 2.0)↑ | 0.0 (-2.0, 3.0)↑ | **0.001** | 0.486 | 0.0 (-2.0, 3.0)↑ | 0.0 (-2.0, 2.0)↑ | **<0.001** | 0.375 | 0.0 (-3.0, 2.0)↑ | 0.0 (-2.0, 3.0)↑ | **<0.001** | **<0.001** |
| **Indicators for evaluating excessive intake——dietary guidelines recommend reducing consumption [median (IQR)]** | | | | | | | | | | | | | |
| **Cooking Oils** | 1.0 (0.0, 3.0)↑ | 1.0 (0.0, 3.6) | 1.0 (0.0, 2.6) | **<0.001** | 0.247 | 1.0 (0.0, 2.6)↑ | 1.0 (0.0, 3.6)↑ | **<0.001** | 0.942 | 1.0 (0.0, 3.2) | 1.0 (0.0, 2.8)↑ | **0.041** | **<0.001** |
| **Addible Sugar** | 0.0 (0.0, 0.0) | 0.0 (0.0, 0.0) | 0.0 (0.0, 0.0) | 0.681 | 0.942 | 0.0 (0.0, 0.0) | 0.0 (0.0, 0.0) | 0.093 | 0.523 | 0.0 (0.0, 0.0) | 0.0 (0.0, 0.0) | 0.123 | 0.394 |
| **Salt** | 2.0 (1.0, 3.7) | 2.2 (1.0, 3.8)↓ | 1.9 (1.0, 3.6)↓ | **0.002** | 0.145 | 1.9 (1.0, 3.5)↓ | 2.2 (1.0, 3.9)↓ | **<0.001** | 0.339 | 2.4 (1.0, 4.2)↓ | 1.8 (1.0, 3.2) | **<0.001** | **<0.001** |
| **Alcoholic Beverage** | 0.0 (0.0, 0.0) | 0.0 (0.0, 0.0) | 0.0 (0.0, 0.0) | **<0.001** | **0.017** | 0.0 (0.0, 0.0) | 0.0 (0.0, 0.0) | **<0.001** | **<0.001** | 0.0 (0.0, 0.0) | 0.0 (0.0, 0.0) | 0.819 | 0.159 |
| **Dietary Diversity and Overall Score [median (IQR)]** | | | | | | | | | | | | | |
| **Diet Variety** | -5.0 (-6.0, -4.0)↑ | -5.0 (-6.0, -4.0)↑ | -5.0 (-6.0, -4.0)↑ | **<0.001** | 0.244 | -5.0 (-6.0, -4.0)↑ | -5.0 (-6.0, -4.0)↑ | **0.039** | 0.942 | -6.0 (-7.0, -5.0)↑ | -4.0 (-5.0, -3.0)↑ | **<0.001** | 0.099 |
| **HBS** | 13.1 (9.3, 16.5) | 13.0 (9.3, 16.5) | 12.8 (8.6, 16.9)↑ | 0.362 | 0.056 | 13.0 (8.8, 17.0)↑ | 13.0 (9.4, 16.3) | 0.860 | 0.158 | 14.6 (11.7, 18.0) | 10.7 (7.6, 14.9)↑ | **<0.001** | **<0.001** |
| **LBS** | 23.0 (17.8, 28.0) | 24.3 (18.7, 29.7)↓ | 23.0 (18.0, 28.6) | **<0.001** | 0.375 | 23.0 (17.6, 28.3) | 24.0 (18.9, 29.4)↓ | **<0.001** | 0.145 | 27.1(22.5, 31.8)↓ | 20.0 (15.5, 24.8) | **<0.001** | **<0.001** |
| **DQD** | 36.5 (28.7, 43.2) | 38.0 (29.5, 44.6)↓ | 36.4 (27.9, 44.2)↑ | **0.005** | 0.105 | 36.5 (28.0, 43.7) | 37.4 (29.3, 44.4) | **0.004** | 0.227 | 42.1 (36.3, 47.9)↓ | 31.1 (24.9, 38.3)↑ | **<0.001** | **<0.001** |

**Note:** Upward arrows indicate that the indicator shows an increasing trend over time, downward arrows indicate a decreasing trend over time, and no arrow indicates that the temporal trend test for the indicator was not statistically significant. P-values represent the results of the difference test, with bolded values indicating statistical significance. P-values for interaction are derived from analysis of variance (ANOVA) examining the interaction between subgroup characteristics and time; bolded values indicate statistically significant interactions.

**Supplementary Table 4** Dietary quality of adults in Beijing across different characteristics, n(%)

|  | **Age** | | |  | **Gender** | |  | **Area** | |  |
| --- | --- | --- | --- | --- | --- | --- | --- | --- | --- | --- |
|  | **18~44 years** | **45~59 years** | **60 years and above** | **p-value** | **Female** | **Male** | **p-value** | **Rural** | **Urban** | **p-value** |
| n | 1,281 | 1,555 | 1,684 |  | 2,452 | 2,068 |  | 2,147 | 2,373 |  |
|  |  |  |  |  |  |  |  |  |  |  |
| **DQD** |  |  |  | 0.919 |  |  | **0.027** |  |  | **<0.001** |
| Good or Acceptable | 38 (3.0) | 45 (2.9) | 71 (4.2) |  | 98 (4.0) | 56 (2.7) |  | 9 (0.4) | 145 (6.1) |  |
| Mild | 535 (41.8) | 591 (38.0) | 704 (41.8) |  | 1016 (41.4) | 814 (39.4) |  | 452 (21.1) | 1378 (58.1) |  |
| Moderate | 610 (47.6) | 760 (48.9) | 732 (43.5) |  | 1119 (45.6) | 983 (47.5) |  | 1322 (61.6) | 780 (32.9) |  |
| Severe | 98 (7.7) | 159 (10.2) | 177 (10.5) |  | 219 (8.9) | 215 (10.4) |  | 364 (17.0) | 70 (2.9) |  |
| **HBS** |  |  |  | 0.352 |  |  | **0.005** |  |  | **<0.001** |
| Good or Acceptable | 377 (29.4) | 464 (29.8) | 547 (32.5) |  | 777 (31.7) | 611 (29.5) |  | 346 (16.1) | 1042 (43.9) |  |
| Mild | 732 (57.1) | 885 (56.9) | 902 (53.6) |  | 1314 (53.6) | 1205 (58.3) |  | 1394 (64.9) | 1125 (47.4) |  |
| Moderate | 171 (13.3) | 201 (12.9) | 234 (13.9) |  | 359 (14.6) | 247 (11.9) |  | 401 (18.7) | 205 (8.6) |  |
| Severe | 1 (0.1) | 5 (0.3) | 1 (0.1) |  | 2 (0.1) | 5 (0.2) |  | 6 (0.3) | 1 (0.0) |  |
| **LBS** |  |  |  | 0.443 |  |  | **0.014** |  |  | **<0.001** |
| Good or Acceptable | 107 (8.4) | 104 (6.7) | 158 (9.4) |  | 220 (9.0) | 149 (7.2) |  | 38 (1.8) | 331 (13.9) |  |
| Mild | 674 (52.6) | 724 (46.6) | 834 (49.5) |  | 1243 (50.7) | 989 (47.8) |  | 768 (35.8) | 1464 (61.7) |  |
| Moderate | 460 (35.9) | 657 (42.3) | 604 (35.9) |  | 889 (36.3) | 832 (40.2) |  | 1174 (54.7) | 547 (23.1) |  |
| Severe | 40 (3.1) | 70 (4.5) | 88 (5.2) |  | 100 (4.1) | 98 (4.7) |  | 167 (7.8) | 31 (1.3) |  |
